# Complete mitochondrial genomes of *Scincella vandenburghi* and *S. huanrenensis* (Squamata: Scincidae)

**DOI:** 10.1080/23802359.2016.1156490

**Published:** 2016-03-28

**Authors:** Jaejin Park, Kyo-Soung Koo, Il-Hun Kim, Daesik Park

**Affiliations:** a Department of Biological Sciences, Kangwon National University, Chuncheon-si, Kangwon-do, South Korea;; b National Marine Biodiversity Institute of Korea, Seocheon-gun, Chungcheongnam-do, South Korea;; c Division of Science Education, Kangwon National University, Chuncheon-si, Kangwon-do, South Korea

**Keywords:** Mitochondrion, *Scincella huanrenensis*, *Scincella vandenburghi*, skink

## Abstract

Here, we report the complete mitochondrial genomes of the skink species *Scincella vandenburghi* and *S. huanrenensis*. The mitogenomes were determined to be 17103 bp for *S. vandenburghi* and 17212 bp for *S. huanrenensis*. The mitogenomes consist of 13 protein-coding genes, two rRNA genes, 22 tRNA genes and two non-coding regions. We then used the mitogenome data to construct a phylogenetic tree for these two species and an additional 16 species within the suborder Lacertilia.

The species *Scincella vandenburghi* is commonly found throughout the Korean peninsula and is present on Tsushima Island, Japan. The distribution of *S. huanrenensis* extends from the northern region of South Korea to the areas of Huanren Co., Liaoning Prov., China (Zhao & Adler 1993; Chen et al. 2001; Lee 2010). Less than 15 populations of *S. huanrenensis* were reported in South Korea (Lee 2010). There are currently no published studies describing the complete mitochondrial genome of any species in genus *Scincella*. Therefore, we determined the complete mitochondrial genomes of both species.

The specimen of *S. vandenburghi* was collected from Yeongwol-gun (N 37°16′55.28″, E 128°23′18.71″), and *S. huanrenensis* was collected from Pyeongchang-gun, Gangwon-do, South Korea (N 37°25′56.69″, E 128°30′27.69″). The samples were preserved in 70% ethanol and deposited at the Herpetology laboratory of Kangwon National University (Voucher nos. G389SV and G390SH). Whole genomic DNA was extracted from the tail tissues of the specimens using the QIAGEN DNeasy Blood & Tissue kit (QIAGEN Korea Ltd, Seoul, South Korea) according to the instructions of the manufacturer. The genomic DNA was sequenced using the Hiseq2000 platform (Illumina Inc., San Diego, CA). We used Geneious 9.0.4 (Biomatters Ltd, Auckland, New Zealand), tRNA Scan-SE1.21 software (http://lowelab.ucsc.edu/tRNA Scan-SE/) and the Dual Organellar GenoMe Annotator to assemble and annotate the mitochondrial DNA sequences.

The mitochondrial genome (GenBank accession no. KU646826) was 17103 bp, and the base composition was 32.0% A, 27.1% T, 14.3% G and 26.7% C in *S. vandenburghi*. There was an A–T rich (59.1%) feature noted in *S. vandenburghi*. The *S. huanrenensis* mitochondrial genome (GenBank accession no. KU507306) was 17212 bp and consisted of 31.7% A, 27.0% T, 14.8% G and 26.5% C. There was also an A–T rich (58.7%) feature observed. Both genomes contained 13 protein-coding genes, two rRNA genes (12S and 16S RNA), 22 tRNA genes and two non-coding regions of an L-strand replication origin and a displacement loop region. The gene arrangement pattern and transcription directions were identical to previous studies in Lacertilia (Kumazawa & Nishida 1999; Kim et al. 2015; Song et al. 2016).

A phylogenetic analysis revealed that there were evolutionary relationships between *S. vandenburghi*, *S. huanrenensis* and an additional 16 lizard species (Figure 1). The two skink species showed a close phylogenetic relationship with other species in Scincidae, which clustered in a monophyletic group. This phylogenetic result is similar to a previous phylogenetic analysis based on the combined nucleotide sequences of nuclear RAG-1 and c-mos in Lacertilia (Townsend et al. 2004).

**Figure 1.**
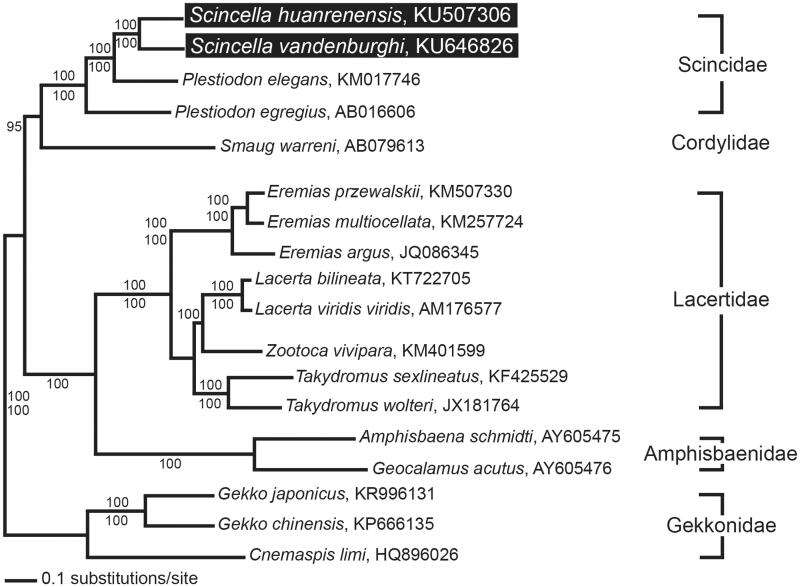
A maximum likelihood (ML) tree based on the complete mitochondrial genomes of *Scincella vandenburghi* and *S. huanrenensis* in addition to 16 other lizard species. The complete mitogenome was downloaded from GenBank using the accession number indicated after the scientific name of each species. The phylogenetic tree was constructed with PAUP* v4.0b10 (Massachusetts Institute of Technology, Cambridge, MA) using 1000 bootstrap replicates. The Bayesian posterior probabilities (above) and bootstrap value (below) are denoted on each branch.
